# Seminal plasma nerve growth factor signaling on the reproductive physiology of female llamas

**DOI:** 10.1590/1984-3143-AR2022-0116

**Published:** 2023-02-13

**Authors:** Luis Paiva, Mauricio Silva, Rodrigo Carrasco, Vicente Ratto, José Goicochea, Marcelo Ratto

**Affiliations:** 1 Escuela de Medicina Veterinaria, Facultad de Agronomía e Ingeniería Forestal, Facultad de Ciencias Biológicas y Facultad de Medicina, Pontificia Universidad Católica de Chile, Santiago, Chile; 2 Departamento de Medicina Veterinaria y Salud Publica, Facultad de Recursos Naturales, Universidad Católica de Temuco, Temuco, Chile; 3 Department of Chemistry, College of Arts and Science, University of Saskatchewan, Saskatoon, Canada; 4 Instituto de Ciencia Animal, Facultad de Ciencias Veterinarias, Universidad Austral de Chile, Valdivia, Chile; 5 Departamento de Cirugía y Biotecnología Reproductiva, Facultad de Medicina Veterinaria y Zootecnia, Universidad Nacional Hermilio Valdizán, Huánuco, Perú

**Keywords:** semen, ovulation-inducing factor, camelids, GnRH

## Abstract

The ovulation mechanism is one of the fascinating physiological processes in reproductive biology in mammals. From the reproductive point of view, the species have been classified as spontaneous or induced ovulators. Although the release of GnRH followed by the preovulatory LH surge is shared between both types of ovulation, the stimulus to initiate GnRH release varies between both categories. In spontaneous ovulators, ovulation depends on the systemic concentration of ovarian steroids, however, in induced ovulators, different stimuli such as copulation, environmental, and social cues can facilitate or induce ovulation regardless of the increases in systemic estradiol concentration. In this review, we document evidence that a male-derived protein is the main factor responsible for inducing ovulation and also modulating the ovarian function in the domestic South American camelid, the llama. The neurotrophin beta-Nerve Growth Factor (β-NGF) is the principal factor present in the semen of llamas responsible for inducing ovulation in this species. After the intrauterine deposit of semen during mating, β-NGF is absorbed through the endometrium to reach the circulatory system, where it reaches the hypothalamus and stimulates GnRH release. The potential site of action of this neurotrophin at the brain has not been elucidated, however, hypotheses are raised that the factor may cross the blood-brain barrier and stimulate upstream neuronal networks that lead to the stimulation of GnRH-secreting neurons. It is possible that β-NGF could be sensed at the median eminence without crossing the blood-brain barrier. Finally, it has been observed that this factor is not only a powerful stimulator of ovulation but also has a luteotrophic effect, resulting in the development of a corpus luteum capable of secreting more progesterone when compared to other ovulation-stimulating analogues.

## Introduction

Seminal plasma (SP) is produced by accessory sex glands (ampulla, vesicular glands, prostate, bulbourethral glands) of the male, and its volume and composition may vary according to the glands present among different species. For many decades SP was considered just a maintenance and transport medium for sperm, but in recent years, it has also been considered as a way of communication between male and female after mating, increasing fertility by improving uterine receptivity and embryo survival (Robertson and Martin, 2022). The importance of accessory glands has been demonstrated by monitoring fecundity alterations following their removal (Pang et al., 1979; Peitz and Olds-Clarke 1986; Wong et al., 2007). Studies in mice and humans have shown that uterine and oviductal changes induced by SP after insemination improve the maternal adaptive immune system during the peri-implantation period, facilitating recognition and tolerance to foreign paternal antigens expressed in the conceptus (Bromfield, 2016).

However, the influence of SP in the female reproductive tract extends beyond local uterine effects. Hormones, neuropeptides and neurotransmitters present in the SP have the potential to affect different organs, including those of the hypothalamic-pituitary-gonadal axis. Early studies have reported that the SP of different species contains nerve growth factor (NGF; Harper and Thoenen, 1980; Harper et al., 1982), which is produced in the vesicle glands (Hofmann and Unsicker, 1982). The extended actions of NGF are clearly exemplified by the neurotrophin that is synthesized in the prostate gland (Bogle et al., 2018) and present in high amounts in the llama SP. Parenteral administration of β-NGF can trigger ovulation in this species (Ratto et al., 2012) and also exerts a potent luteotrophic effect. Thus, the male-derived β-NGF is a key SP protein exerting dramatic actions that can directly modulate the activity of the reproductive axis in females.

In this review, we present our current understanding of how β-NGF present in the SP of males elicits endocrine actions in the female that result in ovulation and luteotrophic effects.

## Mechanisms of ovulation in mammals

Ovulation in mammals relies on the preovulatory GnRH release from the medial basal hypothalamus into the hypophyseal portal system, which triggers an LH surge from the gonadotropes into the systemic circulation, the ultimately elicits the follicular rupture. Based on the stimuli that initiate this process, mammals have been classified as spontaneous or induced ovulatory species. In spontaneous ovulatory species (e.g. cattle, horse, pig), increasing concentrations of systemic estradiol -synthetized from the preovulatory follicle- leads to an increase in the frequency and amplitude of GnRH pulses from the hypothalamus that, when it reaches a certain threshold, triggers the subsequent LH surge (Karsch et al., 1987). In induced ovulators (e.g. rabbit, cats, camelids), the neural signals that result from the physical stimulation of the female reproductive tract caused by the penis during mating have traditionally been considered the main factor associated with the preovulatory discharge of LH and subsequent ovulation (Bakker and Baum, 2000; Kauffman and Rissman, 2006). However, studies conducted in camelids and rabbits have shown the involvement of stimuli other than or in addition to penile intromission as triggers for the ovulatory response, challenging the original conception of mechanical stimulation of the female reproductive tract (Adams et al., 2016; Berland et al., 2016; Maranesi et al., 2018; Ratto et al., 2019).

In South American camelids (llamas and alpacas), Adams et al. (2005) reported that SP contains a potent ovulating-inducing factor (OIF) that later was chemically identified as *β*-NGF (Ratto et al., 2012; Kershaw-Young et al., 2012). This protein is present in the SP of several mammalian species, including cattle and humans (Bogle et al., 2011). Since the discovery of SP β-NGF as the trigger of ovulation in llamas, many investigations have been conducted to elucidate the potential mechanisms of action on the hypothalamus-hypophysis-gonadal axis (Silva et al., 2011, 2012; Carrasco et al., 2018a, b, 2021), and also on the ovary (Valderrama et al., 2019, 2021). It is well established that intramuscular, intravenous, or intrauterine infusion of both purified β-NGF or SP induce a rapid LH surge that is followed by ovulation (Adams and Ratto, 2013; Adams et al., 2016; Silva et al., 2015; Berland et al., 2016) similar to that observed following mating (Bravo et al., 1990). In rabbits, β-NGF may induce ovulation through a hybrid mechanism combining endocrine and nervous components (Maranesi et al., 2018). Therefore, the mechanism of induction of ovulation in rabbits may be different from that observed in llamas (Maranesi et al., 2018) but would also be mediated by β-NGF contained in the SP.

## Endocrine action of β-NGF to control ovulation in llamas

Following the study of Adams et al. (2005), reporting the ovulation and enhanced luteal activity in response to SP administration, subsequent studies in llamas have deepened our understanding of the mechanism involved in these physiological responses. Silva et al. (2011) reported that the magnitude of LH release is reduced when purified β-NGF is injected i.m. in ovariectomized females. The LH peak was restored when these animals were treated with a dose of estradiol benzoate and then challenged with β-NGF. Thus, the effect of β-NGF on the LH peak is influenced by ovarian steroids. Moreover, the preovulatory LH surge induced by β- NGF was abolished when llamas were previously treated with the GnRH antagonist, Cetrorelix (Silva et al., 2012), indicating that the effect of β-NGF on the LH peak is exerted at the hypothalamus by direct or indirect stimulation of GnRH neurons.

The notion that the male controls female ovulation was conceptualized in a study (Berland et al., 2016) in llamas where females were assigned to receive mating with an intact male or with an urethrostomized male (penis intromission but no semen deposition). Ovulations were preceded by an LH surge only in those females mated by the intact male or treated by intrauterine infusion of SP. In the ovulated groups, there was a significant increase in plasma β-NGF that was positively correlated with the LH surge. From this result the following conclusions can be drawn: i) β-NGF is the molecule responsible for ovulation in llamas rather than the mechanical stimuli of the penis in the reproductive tract, as previously thought, and ii) β-NGF is rapidly absorbed through the endometrium into the systemic circulation after mating.

The question of how β-NGF reaches the brain to stimulate GnRH neurons remains unanswered. Ratto et al. (2019) propose two potential pathways by which β-NGF could signal the hypothalamus: i) through crossing the brain-blood barrier at the choroid plexus to reach the cerebral spinal fluid of the ventricular system and activating a neuronal network that results in the release of GnRH or ii) via P75 receptors expressed by the tanycytes -a specialized ependymal cell- which is in contact with circulating β-NGF and play a crucial role in the mechanism of GnRH release (Ojeda et al., 2010).

Since GnRH neurons of the llama hypothalamus do not express the high-affinity receptor for β-NGF, TrkA (Carrasco et al., 2018a), a potential explanation may be that β-NGF acts through kisspeptin neurons in the hypothalamus to stimulate GnRH secretion. In llamas, intravenous administration of kisspeptin elicits LH release and ovulation (Silva et al., 2015), and this effect is consistent with morphological evidence describing close contact between kisspeptin and GnRH neurons in the mediobasal llama hypothalamus (Carrasco et al., 2020, Berland et al., 2021). However, immunohistochemical expression of the β-NGF receptor has not been detected in llama Kisspeptin neurons of the arcuate nucleus or the preoptic area of the hypothalamus (Carrasco et al., 2020).

Further studies will require complex experiments in order to elucidate the mechanism of action of β-NGF at the hypothalamic level using the llama as an animal model.

## The luteotrophic effect of β-NGF in llamas

Administration of SP induces ovulation by eliciting a sustained preovulatory LH surge, which results in a subsequent luteotrophic effect occurring seven days after ovulation, corpora lutea induced by OIF are larger and release 2.5 times more progesterone in comparison to those induced by GnRH administration (Adams et al., 2005). This effect has also been detected in several additional studies (Ratto et al., 2011; Tanco et al., 2011; Silva et al., 2012; Ulloa-Leal et al., 2014). Moreover, the luteal function is enhanced regardless of the size of the preovulatory follicle at the time of treatment with β-NGF (Silva et al., 2014). The positive relationship between the magnitude and extension of the LH release profile and the subsequent luteal function has led to the hypothesis that the luteotrophic effect of llama β-NGF is mediated by changes in LH secretion pattern. Consistent with this notion, Berland et al. (2016) observed that both systemic concentrations of β-NGF and LH increased during the first 3.5 h after mating or intrauterine administration of β-NGF in llamas.

Together with the effect of β-NGF on LH release, it is plausible to propose that β-NGF may also exert a local effect on the ovarian tissue by potentiating progesterone production, as the high-affinity β-NGF receptor, TrkA, has been detected in granulosa cells of preovulatory follicles and also luteal cells of different species (Dissen et al., 1995; Shi et al., 2004; Salas et al., 2006; Carrasco et al., 2016). In llamas, the luteotrophic effect of β-NGF has been associated with enhanced tissue vascularization during the peri-ovulatory period and early stages of CL development, enhancing steroidogenesis (Ulloa-Leal et al., 2014; Fernandez et al., 2014). Furthermore, studies (Silva et al., 2017; Valderrama et al., 2019, 2021) on gene expression of transcripts encoding steroidogenic enzymes related to progesterone production in llamas show upregulation of the steroidogenic acute regulatory protein (StAR), CYP11A1 (P450scc), and 17β hydroxysteroid dehydrogenase (17β HSD) in granulosa cells from preovulatory follicles and also up regulation of StAR and CYP11A1 in luteal cells collected 4 days after ovulation, indicating that β-NGF can promote higher progesterone production in the corpus luteum.

## Conclusions

The evidence presented in this review support that when a male llama mates with a female, β-NGF produced by male accessory sex glands (especially the prostate gland) and present in the SP stimulates the endocrine cascade that induces ovulation and stimulates luteogenesis in the female. This protein is absorbed from the endometrium into the circulatory system, reaching the hypothalamus to trigger GnRH release which is followed by the LH surge. The profile and pattern of LH release induced by β-NGF play an important role in the process of luteogenesis. However, a direct β-NGF effect on the ovary could also potentiate the luteotrophic process. More studies are needed to elucidate the potential sites of action of β-NGF at the hypothalamic-pituitary-ovarian axis.

## References

[B001] Adams GP, Ratto MH, Huanca W, Singh J (2005). Ovulation-inducing factor in the seminal plasma of alpacas and llamas. Biol Reprod.

[B002] Adams GP, Ratto MH, Silva ME, Carrasco RA (2016). Ovulation-inducing factor (OIF/NGF) in seminal plasma: a review and update. Reprod Domest Anim.

[B003] Adams GP, Ratto MH (2013). Ovulation-inducing factor in seminal plasma: a review. Anim Reprod Sci.

[B004] Bakker J, Baum MJ (2000). Neuroendocrine regulation of GnRH release in induced ovulators. Front Neuroendocrinol.

[B005] Berland M, Paiva L, Santander LA, Ratto MH (2021). Distribution of GnRH and kisspeptin immunoreactivity in the female llama hypothalamus. Front Vet Sci.

[B006] Berland M, Ulloa-Leal C, Barría M, Wright H, Dissen GA, Silva ME, Ojeda SR, Ratto MH (2016). Seminal plasma induces ovulation in llamas in the absence of a copulatory stimulus: role of nerve growth factor as an ovulation- inducing factor. Endocrinology.

[B007] Bogle O, Carrasco R, Ratto MH, Singh J, Adams GP (2018). Source and localization of ovulation- inducing factor/nerve growth factor in male reproductive tissues among mammalian species. Biol Reprod.

[B008] Bogle OA, Ratto MH, Adams GP (2011). Evidence for the conservation of biological activity of ovulation-inducing factor in seminal plasma. Reproduction.

[B009] Bravo PW, Fowler ME, Stabenfeldt GH, Lasley B (1990). Endocrine responses in the llama to copulation. Theriogenology.

[B010] Bromfield JJ (2016). A role for seminal plasma in modulating pregnancy outcomes in domestic species. Reproduction.

[B011] Carrasco R, Singh J, Adams GP (2016). The dynamics of trkA expression in the bovine ovary are associated with a luteotrophic effect of ovulation-inducing factor/nerve growth factor (OIF/NGF). Reprod Biol Endocrinol.

[B012] Carrasco RA, Singh J, Adams GP (2018). Distribution and morphology of gonadotropin-releasing hormone neurons in the hypothalamus of an induced ovulator - The llama (*Lama glama*). Gen Comp Endocrinol.

[B013] Carrasco RA, Singh J, Adams GP (2018). The relationship between gonadotropin releasing hormone and ovulation inducing factor/nerve growth factor receptors in the hypothalamus of the llama. Reprod Biol Endocrinol.

[B014] Carrasco RA, Leonardi CE, Hutt K, Singh J, Adams GP (2020). Kisspeptin induces LH release and ovulation in an induced ovulator. Biol Reprod.

[B015] Carrasco RA, Singh J, Ratto MH, Adams GP (2021). Neuroanatomical basis of the nerve growth factor ovulation-induction pathway in llamas. Biol Reprod.

[B016] Dissen GA, Hirshfield AN, Malamed S, Ojeda SR (1995). Expression of neurotrophins and their receptors in the mammalian ovary is developmentally regulated: changes at the time of folliculogenesis. Endocrinology.

[B017] Fernández A, Ulloa-Leal C, Silva M, Norambuena C, Adams GP, Guerra M, Ratto MH (2014). The effect of repeated administrations of llama ovulation-inducing factor (OIF/NGF) during the peri-ovulatory period on corpus luteum development and function in llamas. Anim Reprod Sci.

[B018] Harper GP, Thoenen H (1980). Nerve Growth Factor: Biological Significance, Measurement and Distribution. J Neurochem.

[B019] Harper GP, Glanville RW, Thoenen H (1982). The purification of nerve growth factor from bovine seminal plasma. Biochemical characterization and partial amino acid sequence. J Biol Chem.

[B020] Hofmann HD, Unsicker K (1982). The seminal vesicle of the bull: a new and very rich source of nerve growth factor. Eur J Biochem.

[B021] Karsch FJ, Cummins JT, Thomas GB, Clarke IJ (1987). Steroid feedback inhibition of pulsatile secretion of gonadotropin releasing hormone in the ewe. Biol Reprod.

[B022] Kauffman AS, Rissman EF, Neill JD (2006). The physiology of reproduction, Knobil and Neill’s physiology of reproduction.

[B023] Kershaw-Young CM, Druart X, Vaughan J, Maxwell WMC (2012). β-Nerve growth factor is a major component of alpaca seminal plasma and induces ovulation in female alpacas. Reprod Fertil Dev.

[B024] Maranesi M, Petrucci L, Leonardi L, Piro F, García Rebollar P, Millán P, Cocci P, Vullo C, Parillo F, Moura A, Gonzalez Mariscal G, Boiti C, Zerani M (2018). New insights on a NGF-mediated pathway to induce ovulation in rabbits (*Oryctolagus cuniculus*). Biol Reprod.

[B025] Ojeda SR, Lomniczi A, Sandau U (2010). Contribution of glial-neuronal interactions to the neuroendocrine control of female puberty. Eur J Neurosci.

[B026] Pang SF, Chow PH, Wong TM (1979). The role of the seminal vesicles, coagulating glands and prostate glands on the fertility and fecundity of mice. J Reprod Fertil.

[B027] Peitz B, Olds-Clarke P (1986). Effects of seminal vesicle removal on fertility and uterine sperm motility in the house mouse. Biol Reprod.

[B028] Ratto MH, Berland M, Silva ME, Adams GP (2019). New insights of the role of β-NGF in the ovulation mechanism of induced ovulating species. Reproduction.

[B029] Ratto MH, Delbaere LTJ, Leduc YA, Pierson RA, Adams GP (2011). Biochemical isolation and purification of ovulation-inducing factor (OIF) in seminal plasma of llamas. Reprod Biol Endocrinol.

[B030] Ratto MH, Leduc Y, Valderrama XP, van Straaten K, Delbaere L, Pierson R, Adams GP (2012). The Nerve of Ovulation Inducing Factor. Proc Natl Acad Sci USA.

[B031] Robertson SA, Martin G (2022). Perspective: re-defining “pheromone” in a mammalian context to encompass seminal fluid. Front Vet Sci.

[B032] Salas C, Julio-Pieper M, Valladares M, Pommer R, Vega M, Mastronardi C, Kerr B, Ojeda SR, Lara H, Romero C (2006). Nerve growth factor-dependent activation of trkA receptors in the human ovary results in synthesis of follicle-stimulating hormone receptors and estrogen secretion. J Clin Endocrinol Metab.

[B033] Shi Z, Jin W, Watanabe G, Suzuki AK, Takahashi S, Taya K (2004). Expression of nerve growth factor (NGF), and its receptors trkA and p75 in ovaries of the cyclic golden hamster (Mesocricetus auratus) and the regulation of their production by luteinizing hormone. J Reprod Dev.

[B034] Silva M, Fernández A, Ulloa-Leal C, Adams GP, Berland MA, Ratto MH (2015). LH release and ovulatory response after intramuscular, intravenous, and intrauterine administration of β-NGF of seminal plasma origin in female llamas. Theriogenology.

[B035] Silva M, Recabarren M, Recabarren S, Adams GP, Ratto MH (2012). Ovarian estradiol modulates the stimulatory effect of ovulation-inducing factor (OIF) on pituitary LH secretion in llamas. Theriogenology.

[B036] Silva M, Smulders JP, Guerra M, Valderrama XP, Letelier C, Adams GP, Ratto MH (2011). Cetrorelix suppresses the preovulatory LH surge and ovulation induced by ovulation-inducing factor (OIF) present in llama seminal plasma. Reprod Biol Endocrinol.

[B037] Silva M, Ulloa-Leal C, Norambuena C, Fernández A, Adams GP, Ratto MH (2014). Ovulation- inducing factor (OIF/NGF) from seminal plasma origin enhances Corpus Luteum function in llamas regardless the preovulatory follicle diameter. Anim Reprod Sci.

[B038] Silva M, Ulloa-Leal C, Valderrama XP, Bogle OA, Adams GP, Ratto MH (2017). Nerve growth factor from seminal plasma origin (spβ-NGF) increases CL vascularization and level of mRNA expression of steroidogenic enzymes during the early stage of Corpus Luteum development in llamas. Theriogenology.

[B039] Tanco V, Ratto MH, Lazzarotto M, Adams GP (2011). Dose Response of Female Llamas to Ovulation-Inducing Factor (OIF) from Seminal Plasma. Biol Reprod.

[B040] Ulloa-Leal C, Bogle OA, Adams GP, Ratto MH (2014). Luteotrophic effect of ovulation- inducing factor/nerve growth factor present in the seminal plasma of llamas. Theriogenology.

[B041] Valderrama XP, Silva ME, Goicochea J, Ratto MH (2019). The effect of seminal plasma β-NGF on follicular fluid hormone concentration and gene expression of steroidogenic enzymes in llama granulosa cells. Reprod Biol Endocrinol.

[B042] Valderrama XP, Ulloa-Leal C, Silva ME, Goicochea J, Apichela S, Argañaraz M, Sari L, Paiva L, Ratto VF, Ratto MH (2021). β-NGF stimulates steroidogenic enzyme and VEGFA gene expression, and progesterone secretion via ERK 1/2 pathway in primary culture of llama granulosa cells. Front Vet Sci.

[B043] Wong CL, Lee KH, Lo KM, Chan OC, Goggins W, Chow PH (2007). Ablation of paternal accessory sex glands imparts physical and behavioural abnormalities to the progeny: an in vivo study in the golden hamster. Theriogenology.

